# The role of embodied musical engagement in enhancing non-cognitive skills and rehabilitation outcomes in children with special needs

**DOI:** 10.3389/fpsyg.2026.1798176

**Published:** 2026-05-25

**Authors:** Yanyan Lu, Laia Viladot, Xiaoguang Chen, Yue Fan, Jingyi Yuan

**Affiliations:** 1 Department of Musicology, School of Music and Dance, Nanjing Normal University of Special Education, Nanjing, China; 2 Department of Teaching Musical, Artistic and Corporal Expression, Universitat Autònoma de Barcelona, Bellaterra, Spain; 3 Department of Musicology, School of Music, Nanjing Xiaozhuang University, Nanjing, China; 4 The Second Hospital of Nanjing, Affiliated to Nanjing University of Chinese Medicine, Nanjing, China; 5 UCL Institute of Education, University College London, London, United Kingdom

**Keywords:** attention control, children with special needs, embodied musical engagement, non-cognitive skills, rehabilitation outcomes, rhythmic entrainment, structural equation modeling

## Abstract

This study explores the impact of embodied musical engagement on non-cognitive skills and rehabilitation outcomes in children with special needs. A sample of 294 children was recruited (68.7% male; largest age subgroup: 7–9 years, 33.7%) for this investigation. Using Structural Equation Modeling (SEM), the study examined the direct effects of musical engagement on rehabilitation performance and the mediating role of non-cognitive skills—such as attention control, emotion regulation, task persistence, and cooperative behavior. Results revealed that embodied musical engagement had a significant direct effect on rehabilitation performance (*β* = 0.527, *p* < 0.001) and a robust positive influence on non-cognitive skills (β = 0.755, p < 0.001). Mediation analysis confirmed a partial mediation effect, with non-cognitive skills accounting for 34.3% of the total effect (Indirect Effect = 0.275, 95% CI [0.181, 0.376]). This research provides new theoretical insights and empirical evidence for music-based interventions offering practical guidance for the rehabilitation of children with special needs, particularly in the contexts of ASD, ADHD, and intellectual disabilities.

## 1 Introduction

Recent advancements in special education and rehabilitation sciences have highlighted rehabilitation performance as a critical outcome for children with special needs, including those with Autism Spectrum Disorder (ASD), Attention-Deficit/Hyperactivity Disorder (ADHD), and intellectual disabilities (Gao et al., 2025). Rehabilitation performance is commonly reflected in children’s functional participation, behavioral regulation, attention maintenance, and adaptive engagement (World Health Organization, 2001) within structured learning and therapeutic contexts. Improving these outcomes remains a central challenge in both educational and clinical practice, as traditional interventions often yield uneven or short-term effects (Geretsegger et al., 2022).

Among emerging intervention approaches, music-based activities have received increasing attention due to their multimodal nature, integrating auditory, rhythmic, and motor elements that can simultaneously engage multiple sensory systems (Geretsegger et al., 2022). Unlike purely cognitive or verbal interventions, music-based engagement allows children to interact with therapeutic content through bodily movement and rhythmic synchronization. However, existing research has primarily focused on the effectiveness of music interventions at the outcome level, with limited investigation into the underlying mechanisms through which musical engagement translates into improved rehabilitation performance (Geretsegger et al., 2014).

From a theoretical perspective, embodied cognition posits that cognitive and emotional processes are deeply grounded in bodily action and sensorimotor experiences (Srinivasan and Bhat, 2013). Bodily engagement, particularly through rhythmic and coordinated movement, has been shown to influence attention regulation, emotional stability, motivation, and social interaction—capacities commonly conceptualized as non-cognitive skills (Bharathi et al., 2019; Blair and Raver, 2014; Kim et al., 2009).

These skills are widely recognized as foundational mechanisms supporting children’s sustained participation and functional performance in rehabilitation contexts. Accordingly, embodied musical engagement may enhance rehabilitation outcomes not only through direct sensorimotor stimulation, but also indirectly by fostering the development of non-cognitive skills that support adaptive behavior and learning.

## 2 Theoretical analysis and hypothesis development

### 2.1 Theoretical basis of embodied engagement in music activities

Embodied engagement in music activities refers to the process by which individuals actively participate in music through the integration of bodily movements, rhythmic synchronization, postural adjustments, sensory feedback, and emotional experiences (Geretsegger et al., 2022). Embodied cognition theory emphasizes that cognition does not occur solely in the brain but arises from continuous interaction between the body and the environment through sensorimotor loops (Srinivasan and Bhat, 2013).

In musical environments, children are encouraged to engage through simple actions like clapping, swaying, or imitating rhythmic movements (Geretsegger et al., 2022; Sharda et al., 2018). These activities create opportunities for basic sensorimotor coupling, which can support foundational development across neural, emotional, and social domains (Bharathi et al., 2019). From a sensorimotor perspective, rhythmic entrainment requires children to continuously adjust their movements to match external rhythms, thereby engaging neural systems related to motor control, sustained attention, and executive functioning and promoting neural plasticity (Bharathi et al., 2019; Tryfon et al., 2017). In rehabilitation contexts for children with special needs, embodied musical engagement shows distinct advantages: it is intrinsically motivating, reducing barriers to participation for children with ASD or ADHD; it relies primarily on action rather than language, allowing children with delayed language development to engage meaningfully through nonverbal interaction; and it integrates multiple sensory and emotional modalities, providing richer and more effective stimulation than single-modality training (Geretsegger et al., 2022). Accordingly, embodied musical engagement can be conceptualized not only as a form of intervention but also as a core mechanism underlying rehabilitation outcome, which provides a theoretical foundation for the proposed hypotheses. Rhythmic and musical engagement activates distributed auditory–motor networks including the basal ganglia, supplementary motor area, and cerebellum (Zatorre et al., 2007; Chen et al., 2008), supporting motor coordination and timing. Furthermore, rhythmic entrainment facilitates sensorimotor synchronization and executive control processes (Thaut et al., 2015; Bharathi et al., 2019). These mechanisms remain functional and can be leveraged for neuroplastic reorganization in neurodevelopmental conditions such as ASD, where music-based interventions have been shown to enhance auditory–motor connectivity, social communication, and timing skills (Sharda et al., 2018; LaGasse et al., 2024).

### 2.2 Development mechanism of non-cognitive skills

Non-cognitive skills, including emotional regulation, attention control, executive function, self-efficacy, motivation, and social competence—exert enduring influences on children’s development and exhibit distinctive plasticity and developmental trajectories compared with cognitive abilities (Gutman and Schoon, 2016). These skills are highly malleable in childhood, shaped strongly by environmental input and interactive experiences; they exert cross-domain effects on emotional functioning, interpersonal behavior, learning motivation, task persistence, and behavioral adaptability; and their development is fundamentally experience-dependent, relying on emotional interaction and bodily engagement rather than purely cognitive training, with musical activities providing especially rich experiential contexts (Yum et al., 2024). In rehabilitation for children with special needs, non-cognitive skills critically determine engagement, therapeutic alliance, persistence, and long-term adaptive outcomes. Nevertheless, existing research often emphasizes observable performance indicators while overlooking these skills as underlying mechanisms (Gutman and Schoon, 2013). Accordingly, the present study conceptualizes non-cognitive skills as a key mediating factor linking embodied musical engagement to rehabilitation outcomes.

### 2.3 Theoretical pathway: how embodied musical engagement enhances rehabilitation outcomes

Embodied musical engagement can improve rehabilitation outcomes in children with special needs by fostering multi-channel sensorimotor integration, thereby influencing emotion management, attention regulation, social communication, and behavioral adaptability (LaGasse et al., 2024).

First, from the perspective of sensorimotor integration, rhythmic movements, bodily imitation, and musical interactions activate brain circuits linked with motor coordination, executive functions, and pre-linguistic skills, consequently fostering motor abilities and task-control capacities (Srinivasan and Bhat, 2013). For children with ASD or ADHD, embodied rhythmic activities particularly improve bodily coordination and behavioral predictability, reducing unstable behaviors and boosting the quality of rehabilitation participation (Sharda et al., 2018).

Second, embodied musical interactions aid in emotional regulation (Schiavio et al., 2017). Rhythmic synchrony and coordinated movements foster affective synchronization, helping children develop a sense of security, predictability, and social connection (Trainor and Cirelli, 2015). This is especially important for children who are sensitive to emotional stimuli or have low social motivation. Synchronous actions within musical interventions are believed to bolster emotional balance, enabling children to engage more readily in helpful conduct.

**Figure 1:**
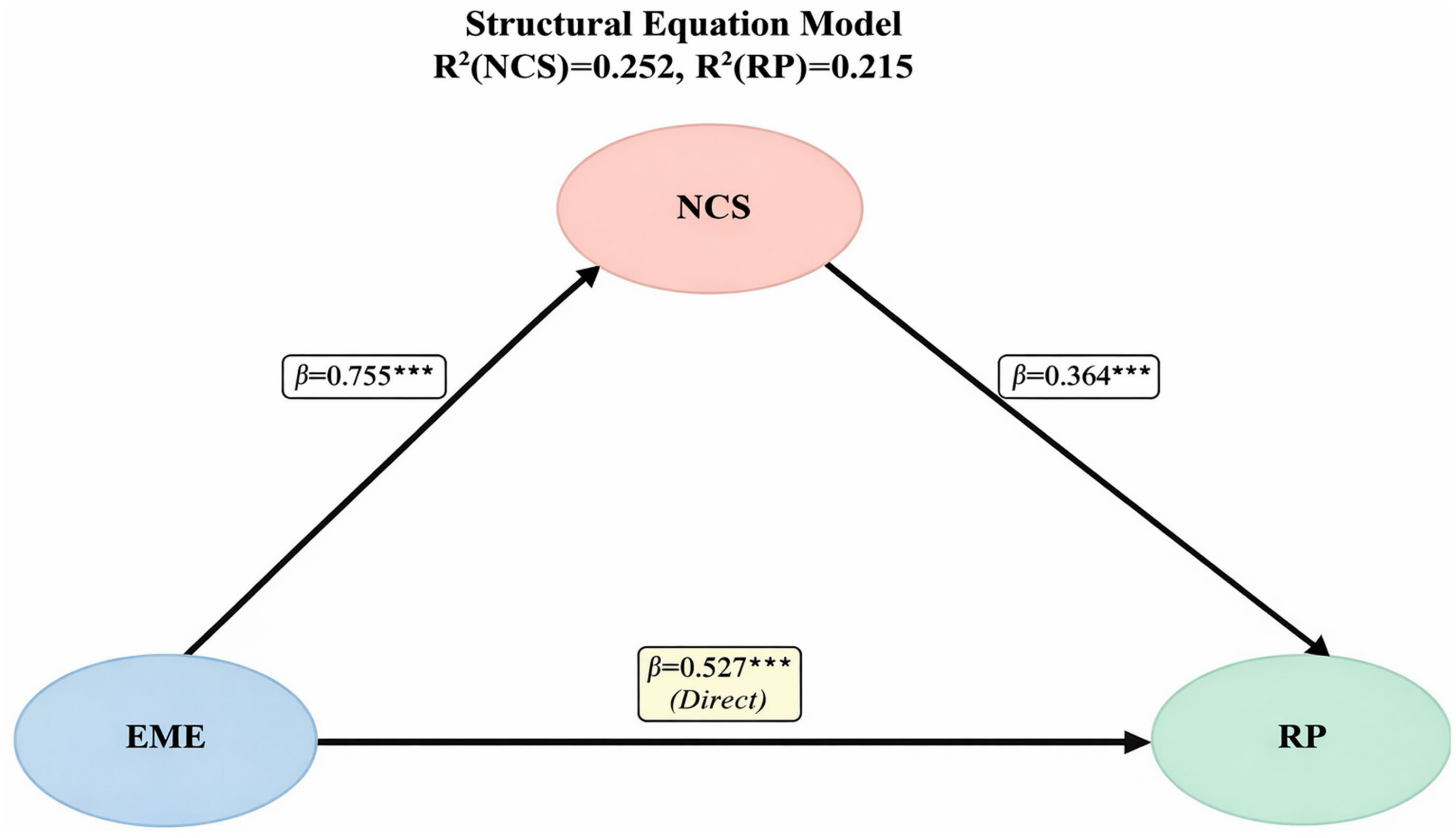
Structural model with standardized path coefficients.

Third, embodied engagement lowers language and cognitive barriers (Kosmas and Zaphiris, 2019). For children with delayed language development or cognitive impairments, music activities based on body movements, rhythm, and gestures do not depend on intricate verbal rules, making it easier to elicit active participation. This leads toward increased frequency and ongoing rehabilitation interactions. Previous research indicates that engagement is one of the strongest predictors of rehabilitation outcomes, and embodied musical engagement is an essential means to enhance it. To summarize, embodied musical engagement promotes rehabilitation outcomes through multiple mechanisms—including behavioral, emotional, motor, and interactive pathways (Figure 1).

### 2.4 Mediating role of non-cognitive skills

Non-cognitive skills may play a critical mediating role in the relationship between embodied musical engagement and rehabilitation outcomes. Embodied musical activities provide a natural context for the development of non-cognitive skills. Long-term engagement within embodied musical activities can improve these key non-cognitive skills, which are directly linked to rehabilitation outcomes. For instance, children with more stable attention are better able to persist with rehabilitation tasks; those with stronger emotional regulation exhibit fewer negative behaviors and higher cooperation during therapy. Thus, improvements in non-cognitive skills may constitute the underlying mechanism through which embodied musical engagement produces rehabilitative effects (Shi et al., 2024).

### 2.5 Research model and hypotheses

Based on the theoretical analysis presented above, this study proposes the following research model to illustrate how embodied musical engagement influences rehabilitation outcomes through non-cognitive skills:

*H1*: Embodied musical engagement has a significant positive effect on rehabilitation outcomes.

*H2*: Embodied musical engagement has a significant positive effect on non-cognitive skills.

*H3*: Non-cognitive skills have a significant positive effect on rehabilitation outcomes.

*H4*: Non-cognitive skills mediate the relationship between embodied musical engagement and rehabilitation outcomes.

## 3 Materials and methods

### 3.1 Participants and data collection

Participants were recruited through a multi-center approach that included rehabilitation centers, special education schools, and collaborating hospitals. To ensure scientific rigor, a stringent screening process was implemented. Of the more than 360 candidates initially invited, 294 met the data validity criteria and provided complete informed consent. In this final sample, 68.7% were male, and the largest age subgroup was 7–9 years old (33.7%), followed by 10–12 years (30.3%), 4–6 years (23.1%), and 13–15 years (12.9%). Data were collected from respondents familiar with the children, including parents (47.6%), rehabilitation therapists (21.1%), grandparents (18.7%), and other primary caregivers (12.6%). In terms of training frequency, 20.4% of children participated 1 time per week, 49.0% 2–3 times per week, and 30.6% 4 or more times per week.

As shown in Table 1, the sample was designed to reflect the ecological diversity in special education settings. In this study, children with special needs are defined as a heterogeneous population with varied developmental profiles. The sample primarily comprises individuals with high-prevalence neurodevelopmental disorders: autism spectrum disorder (ASD, 34.7%), attention-deficit/hyperactivity disorder (ADHD, 25.9%), and intellectual disability (22.1%). It also includes children with hearing impairment (7.8%), physical disability (5.8%), and emotional and behavioral disorders (3.7%). Focusing the analysis on high-prevalence functional disorders ensures adequate statistical power for Structural Equation Modeling (SEM). This diverse sample covers the key populations targeted by embodied musical intervention research: embodied musical interaction design has been specifically validated for children with autism (Ragone, 2020), musical interaction supports children with severe or profound intellectual and multiple disabilities (Johnels et al., 2023), and such experiences align with Eco neurobiological principles that shape child brain development and behavioral outcomes (Mualem et al., 2024).

**Table 1:** Baseline characteristics of the study sample (*N* = 294).

Variable	Category	*n* (%)
Child demographics		
Gender	Male	202 (68.7)
	Female	92 (31.3)
Age group	Preschool (4–6 years)	68 (23.1)
Child age	Early school age (7–9 years)	99 (33.7)
	Pre-adolescence (10-12 years)	89 (30.3)
	Adolescence (13–15 years)	38 (12.9)
Primary clinical diagnosis		
	Autism spectrum disorder	102 (34.7)
	ADHD	76 (25.9)
	Intellectual disability	65 (22.1)
	Hearing impairment	23 (7.8)
	Physical disability	17 (5.8)
	Emotional disorder	11 (3.7)
Informant characteristics		
Respondent role	Parent	140 (47.6)
	Rehabilitation therapists	62 (21.1)
	Grandparents	55 (18.7)
	Other primary caregivers	37 (12.6)
Intervention profile		
Training frequency	1 time/week	60 (20.4)
	2–3 times/week	144 (49.0)
	4 + times/week	90 (30.6)

The study employed a non-interventional, cross-sectional observational design. The primary objective was to examine current relational mechanisms rather than to assess immediate clinical efficacy. Given this cross-sectional observational design, the current study did not establish experimental or control groups, nor did it include post-intervention follow-up; rather, its primary focus remained on exploring the underlying mechanisms.

Table 1 further shows that children with special needs constitute a heterogeneous group with diverse developmental, behavioral, and functional differences. Typically, these atypical developmental trajectories or functional challenges are initially observed by primary caregivers or educators in daily settings, subsequently leading to formal clinical evaluations. In this study, participants were categorized based on their primary clinical diagnoses confirmed by medical professionals, which include autism spectrum disorder (ASD), attention-deficit/hyperactivity disorder (ADHD), intellectual disability, hearing impairment, physical disability, and emotional and behavioral disorders.

Ethics/consent: All participants and their guardians provided informed consent. The study protocol was approved by the relevant hospital or educational institution ethics committee (IRB/Ethics Committee).

### 3.2 Research design

This study employed a cross-sectional survey design to examine the relationships among embodied musical engagement, non-cognitive skills, and rehabilitation performance in children with special needs. Data were collected using standardized questionnaires completed by caregivers, teachers, and rehabilitation therapists who were familiar with the children’s participation in music-based activities and rehabilitation programs. Structural equation modeling (SEM) with bootstrapping was conducted to test the hypothesized mediation pathways among the study variables. Given the cross-sectional nature of the data, the observed relationships should be interpreted as associational rather than causal.

### 3.3 Measures/instruments

#### 3.3.1 Embodied musical engagement

In this study, Embodied Musical Engagement (EME) was operationalized as the children’s observable bodily involvement in music-based activities provided by their rehabilitation centers. These activities typically included rhythmic movements (e.g., clapping, tapping, drumming), whole-body engagement (e.g., swaying, dancing, moving to music), action imitation, simple instrument playing, and interactive music games. The measurement focused on the functional participation of the children in these activities rather than the duration or specific structure of the program.

Given the absence of a standardized instrument specifically designed to assess embodied musical engagement in children with special needs, this study employed a structured composite measurement based on behavioral indicators and multi-informant ratings (Leman, 2007; Phillips-Silver and Trainor, 2005; Srinivasan and Bhat, 2013). The EME construct comprised four dimensions: rhythmic entrainment, physical engagement, imitative action, and interactive participation (Phillips-Silver and Trainor, 2005; Leman, 2007). Items assessed behaviors such as synchronizing body movements with musical rhythms, engaging in simple motor responses (e.g., clapping or swaying), imitating musical actions, and participating in basic turn-taking during musical activities. All items were rated on a 5-point Likert scale ranging from 1 (“never”) to 5 (“always”), based on reports from parents, teachers, and rehabilitation therapists who were familiar with the children’s regular participation in music-based interventions. Inter-rater reliability was examined using the two-way random effects model for single measures of absolute agreement, ICC (2,1) (Shrout and Fleiss, 1979; Koo and Li, 2016). ICC values across parent, teacher, and therapist ratings ranged from 0.82 to 0.89 for all subscales, indicating excellent consistency.

Prior to analysis, internal consistency reliability was assessed. As reported in the Results section, the overall EME scale demonstrated satisfactory reliability (Composite Reliability = 0.876), with Cronbach’s *α* values for the four subdimensions ranging from 0.853 to 0.881, indicating adequate internal consistency for subsequent structural equation modeling.

As detailed in Table 2, the embodied music engagement activities in this study followed a structured protocol. Rhythmic synchronization included clapping, body percussion, and swaying to a steady beat, with the purpose of promoting sensorimotor synchronization and body coordination. Physical engagement involved drumming, stepping, and full-body movements to music to enhance active physical participation and reduce passivity. Imitative action required imitating simple gestures and movements demonstrated by instructors, aiming to facilitate action learning and imitation through modeling. Interactive participation consisted of turn-taking games, passing instruments, and group singing to foster basic social interaction and cooperative skills. Auditory engagement used familiar children’s songs to support engagement through well-known auditory cues. Visual and multisensory support incorporated picture-book music storytelling combined with rhythm singing and visual prompts, providing concrete visual support for children with attention difficulties. The music materials primarily included familiar children’s songs, steady-beat rhythm tracks, and multisensory picture-book music stories. Considering individual differences among children with special needs and the multicenter cross-sectional observational design adopted in this study, specific musical pieces varied across different rehabilitation centers. These activities were naturally implemented by trained therapists as part of routine rehabilitation curricula, usually lasting 30–45 min per session, 1–4 times per week. As previously noted, given that this study employed a cross-sectional observational design without a controlled intervention, no post-intervention follow-up was conducted to assess long-term therapy outcomes. All rehabilitation outcomes were based on cross-sectional assessments conducted at a single time point.

**Table 2:** Embodied music engagement protocol.

Category	Specific activities	Purpose/Rationale
Rhythmic synchronization	Clapping, body percussion, swaying to a steady beat	Promote sensorimotor synchronization and body coordination
Physical engagement	Drumming, stepping, full-body movements to music	Enhance active physical participation and reduce passivity
Imitative action	Imitating simple gestures and movements from instructors	Facilitate action learning and imitation through modeling
Interactive participation	Turn-taking games, instrument passing, group singing	Foster basic social interaction and cooperative skills
Auditory engagement	Familiar children’s songs	Support engagement via well-known auditory cues
Visual & multisensory support	Picture-book music storytelling (rhythm singing + visual prompts)	Provide concrete visual support for children with attention difficulties

The above activities represent core components of routine music rehabilitation programs in participating centers and special education schools. Sessions were delivered by trained therapists following institutional curricula (30–45 min/session, 1–4 times/week). Specific musical pieces differed across centers, but structural elements remained consistent with the categories above.

#### 3.3.2 Non-cognitive skills

Non-cognitive skills were explicitly operationalized as four core, theoretically grounded domains: emotion regulation, attention control, task persistence, and cooperative behavior. Each dimension was defined based on observable, contextually appropriate behaviors in rehabilitation settings. Non-cognitive skills were evaluated based on observable regulatory and task-related behaviors, with emphasis on effort, responsiveness, and recovery rather than sustained or independent performance (Michel and Roebers, 2008). This approach reflects the functional realities of children with developmental disabilities, for whom brief attention, partial emotional regulation, and supported persistence constitute meaningful developmental indicators.

In this study, these skills were assessed using a structured multi-informant report tool; items were adapted from established behavioral and emotional regulation scales and rated by parents, teachers, and therapists familiar with the child’s daily functioning (Gioia and Isquith, 2011; Shields and Cicchetti, 1997).

#### 3.3.3 Rehabilitation outcomes

Rehabilitation outcomes were operationalized as four functionally relevant dimensions: engagement, task persistence, behavioral improvement, and social adaptive skills. All dimensions were assessed via observable behavioral changes in daily rehabilitation participation, ensuring the measurement was grounded in functional practicality and consistent with the study’s theoretical framework. Rehabilitation outcomes were defined in terms of functional participation and observable behavioral change, rather than mastery or optimal performance. Assessment focused on children’s ability to engage, tolerate task demands, and demonstrate gradual improvement within structured rehabilitation contexts. Observable behavioral change was defined as consistent, repeated improvements in attention, emotional stability, cooperation, and task completion. A ≥ 1-point increase on the 5-point Likert scale was used as the threshold for meaningful change, consistent with clinical conventions for children with special needs.

For the purpose of this study, rehabilitation outcomes were operationalized through a composite measure assessing four dimensions: engagement, persistence, behavioral improvement, and social skills, as reported by familiar caregivers and therapists.

### 3.4 Data processing and statistical analysis

Data processing and statistical analyses were conducted using SPSS 26.0 and AMOS 24.0. Prior to hypothesis testing, the psychometric properties of the scales were examined, including tests for reliability, validity, and common method bias. Common method bias was assessed using Harman’s single-factor test and the common latent factor (CLF) approach. Harman’s test showed that the first factor accounted for 35.85% of the total variance, which is below the recommended threshold of 40%. In addition, the CLF analysis indicated that the average change in standardized factor loadings was 0.020, with a maximum change of 0.047, both well under the suggested cutoff of 0.20. These results suggest that common method bias is unlikely to materially affect the findings.

Internal consistency reliability was assessed using Cronbach’s *α* and composite reliability indices.

Confirmatory factor analysis (CFA) was conducted to assess the measurement model. Convergent validity was established using average variance extracted (AVE) and composite reliability, while discriminant validity was examined using the Fornell-Larcker criterion. The model fit was evaluated using multiple indices: the chi-square to degrees of freedom ratio (χ^2^/df), the Comparative Fit Index (CFI), the Tucker–Lewis Index (TLI), the Root Mean Square Error of Approximation (RMSEA), and the Standardized Root Mean Square Residual (SRMR).

Structural equation modeling (SEM) was then applied to test the hypothesized relationships among Embodied Musical Engagement, Non-cognitive Skills, and Rehabilitation Performance. The significance of the hypothesized paths was tested using maximum likelihood estimation. Finally, to examine the mediating role of non-cognitive skills, a bootstrap procedure with 5,000 resamples was employed to estimate the indirect effects and their corresponding 95% confidence intervals.

## 4 Results

### 4.1 Descriptive statistics and correlations

As shown in Table 3, descriptive statistics indicated that children demonstrated moderate levels of embodied musical engagement (M = 3.84, SD = 0.72) and non-cognitive skills (M = 3.24, SD = 1.08). Analysis at the dimensional level showed that rhythmic entrainment and physical engagement yielded relatively higher scores, suggesting that sensorimotor synchronization is more accessible to this population than complex social interaction.

**Table 3:** Descriptive statistics for primary variables and dimensions.

Construct/Dimension	Mean	SD	Min	Max
EME (Overall)	3.84	0.72	2.17	5.00
Rhythmic entrainment	3.86	0.80	1.67	5.00
Physical engagement	4.00	0.79	1.67	5.00
Imitative action	3.71	0.80	1.33	5.00
Interactive participation	3.80	0.82	1.67	5.00
NCS (overall)	3.24	1.08	1.00	5.00
Emotion regulation	3.29	1.17	1.00	5.00
Attention control	3.18	1.20	1.00	5.00
Task persistence	3.33	1.15	1.00	5.00
Cooperative behavior	3.16	1.22	1.00	5.00
RP (overall)	3.24	1.11	1.00	5.00
Engagement	3.32	1.22	1.00	5.00
Persistence	3.29	1.20	1.00	5.00
Behavioral improvement	3.24	1.16	1.00	5.00
Social skills	3.11	1.23	1.00	5.00

As presented in Table 4, Pearson correlation analyses revealed moderate and statistically significant associations among all constructs. Embodied Musical Engagement was positively correlated with Non-cognitive Skills (r = 0.502, *p* < 0.001) and Rehabilitation Performance (r = 0.435, *p* < 0.001). Additionally, Non-cognitive Skills showed a significant positive correlation with Rehabilitation Performance (r = 0.356, *p* < 0.001). Consistent with prior evidence that early music participation promotes children’s non-cognitive and socioemotional development (Gaudette-Leblanc et al., 2021; Li, 2025), these moderate correlations indicate that while the constructs are functionally related, they represent distinct theoretical domains and do not raise multicollinearity concerns. We adopted the threshold of r < 0.70, a widely accepted criterion in structural equation modeling for assessing multicollinearity (Hair et al., 2022). To further verify the robustness of the results, we also examined stricter multicollinearity thresholds (e.g., r < 0.60, r < 0.50), and all inter-construct correlations remained below these cutoffs. These findings confirm that the conclusion of no severe multicollinearity is consistent and robust across different threshold standards, thereby strengthening the reliability of the subsequent structural model analysis.

**Table 4:** Pearson correlation matrix for main constructs.

Variables	EME	NCS	RP
EME	1.000		
NCS	0.502***	1.000	
RP	0.435***	0.356***	1.000

***p < 0.001.

### 4.2 Measurement model assessment

Confirmatory Factor Analysis (CFA) was conducted to evaluate the reliability and validity of the measurement model for the three latent constructs: Embodied Musical Engagement (EME), Non-cognitive Skills (NCS), and Rehabilitation Performance (RP).

Reliability and Convergent Validity The measurement model demonstrated excellent psychometric properties. Internal consistency reliability was examined for the three latent variables. As presented in Table 5, Composite Reliability (CR) values ranged from 0.853 to 0.901, well above the recommended threshold of 0.70 (Hair et al., 2022). Convergent validity was established as all standardized factor loadings ranged from 0.724 to 0.845 (*p* < 0.001), significantly exceeding the 0.70 benchmark. The Average Variance Extracted (AVE) values were between 0.581 and 0.647, exceeding the 0.50 cutoff, confirming satisfactory convergent validity.

**Table 5:** Convergent validity: composite reliability and average variance extracted.

Construct/Dimension	Items	CR	AVE
EME (Overall)	12	0.876	0.608
Rhythmic entrainment	3	0.869	0.591
Physical engagement	3	0.881	0.614
Imitative action	3	0.853	0.581
Interactive participation	3	0.871	0.595
NCS (overall)	12	0.901	0.637
Emotion regulation	3	0.893	0.625
Attention control	3	0.908	0.647
Task persistence	3	0.889	0.619
Cooperative behavior	3	0.897	0.631
RP (overall)	12	0.887	0.619
Engagement	3	0.895	0.627
Persistence	3	0.878	0.607
Behavioral improvement	3	0.885	0.616
Social skills	3	0.892	0.623

Discriminant Validity Discriminant validity was evaluated using the Fornell–Larcker criterion. As shown in Table 6, the square roots of the AVE values (bold diagonal elements) were greater than the correlations with other constructs. All inter-construct correlations were statistically significant (*p* < 0.001) but moderate in magnitude (ranging from 0.435 to 0.502), indicating adequate discriminant validity among the three latent variables.

**Table 6:** Discriminant validity assessment using Fornell-Larcker criterion.

Construct	EME	NCS	RP
EME	0.780		
NCS	0.502	0.798	
RP	0.435	0.356	0.787

### 4.3 Structural model analysis

The structural model was tested to examine the hypothesized relationships between Embodied Musical Engagement (EME), Non-cognitive Skills (NCS), and Rehabilitation Performance (RP). The model showed a good fit to the data (see Table 7): χ^2^/df = 1.746, CFI = 0.947, TLI = 0.945, and RMSEA = 0.0572 (Hu and Bentler, 1999).

**Table 7:** Measurement model fit indices.

Fit index	Value	Threshold	Assessment
χ^2^	1089.45	—	—
df	624	—	—
χ^2^/df	1.746	< 3.0	Good
CFI	0.947	> 0.95	Acceptable
TLI	0.945	> 0.95	Acceptable
RMSEA	0.057	< 0.05	Acceptable
SRMR	0.051	< 0.08	Good

The chi-square to degrees of freedom ratio (χ^2^/df = 1.746) fell well below the threshold of 3.0. The Comparative Fit Index (CFI = 0.947) and Tucker-Lewis Index (TLI = 0.945) both approached the stringent 0.95 criterion. The Root Mean Square Error of Approximation (RMSEA = 0.057) was close to the 0.05 threshold.

As detailed in Table 8, the path analysis provided strong empirical support for the proposed hypotheses:

H1 (Direct Effect): Supported. Embodied Musical Engagement had a significant direct positive effect on Rehabilitation Performance (*β* = 0.527, t = 20.007, *p* < 0.001).
H2 (Mediation Path): Supported. Embodied Musical Engagement showed a robust positive impact on Non-cognitive Skills (β = 0.755, t = 40.060, *p* < 0.001).
H3 (Path to Outcome): Supported. Non-cognitive Skills were found to have a significant positive effect on Rehabilitation Performance (β = 0.364, t = 13.544, *p* < 0.001).

**Table 8:** Standardized path coefficients and hypothesis test results.

Hypothesis	Path	β	SE	t-value	*p*-value	Result
H1	EME → RP	0.527	0.026	20.007	< 0.001	Supported
H2	EME → NCS	0.755	0.019	40.060	< 0.001	Supported
H3	NCS → RP	0.364	0.027	13.544	< 0.001	Supported

The model demonstrated moderate to good explanatory power, accounting for 25.2% of the variance in Non-cognitive Skills (*R*^2^ = 0.252) and explaining substantial variance in Rehabilitation Performance through both direct and indirect pathways.

The mediating effect of non-cognitive skills was tested using the bootstrap method with 5,000 resamples (Preacher and Hayes, 2008). As shown in Table 9, the indirect effect of Embodied Musical Engagement → Non-cognitive Skills → Rehabilitation Outcomes was significant (Indirect Effect = 0.275, 95% CI [0.181, 0.376]), confirming H4.

**Table 9:** Mediation analysis: bootstrap results and effect decomposition.

Effect type	Estimate	95% CI Lower	95% CI Upper	Significance
Direct effect	0.527	—	—	*p* < 0.001
Indirect effect	0.275	0.181	0.376	Significant
Total effect	0.801	—	—	*p* < 0.001
Proportion mediated	34.3%	—	—	—

The analysis revealed a partial mediation model: Non-cognitive skills accounted for 34.3% of the total effect, while 65.7% of the effect was direct. These findings suggest a dual-pathway mechanism where embodied engagement influences rehabilitation both directly through sensorimotor activation and indirectly by fostering non-cognitive capabilities.

## 5 Discussion of results

This study applied Structural Equation Modeling (SEM) to test the theoretical pathway of “Embodied Musical Engagement → Non-cognitive Skills → Rehabilitation Outcomes,” and the core findings are interpretable and supported by existing literature as well as embodied cognition theory. These findings suggest that embodied musical engagement functions not merely as a therapeutic activity, but as a structured developmental context that supports self-regulation and adaptive functioning.

The present findings are consistent with recent clinical evidence from (Liu and Huang, 2025a, b) supporting music therapy as an effective modality for improving behavioral and functional outcomes in children with neurodevelopmental disorders. Music-based interventions that emphasize rhythmic synchronization and embodied participation have been shown to enhance engagement, social communication, and adaptive behaviors, particularly for children with autism spectrum disorder and related conditions. Recent meta-analyses and randomized controlled trials further consolidate this evidence base: (Gao et al., 2025) systematically verified that music therapy significantly alleviates core behavioral symptoms in autistic children, with robust pooled effect sizes across multiple standardized assessment scales; (Kanzari et al., 2025) demonstrated that structured music-and-movement programs concurrently improve motor competence, social engagement, and adaptive behavior, providing direct empirical support for the multi-domain benefits of embodied musical activities; (Navarro et al., 2025) conducted an updated meta-analysis confirming that music interventions enhance social interaction, attention control, and emotional stability in autistic populations, aligning with the non-cognitive skill dimensions measured in this study; and (Goes et al., 2025) provided targeted evidence for children with ADHD, showing that music-based interventions reduce hyperactivity-impulsivity and improve sustained attention, reinforcing the generalizability of the present findings across distinct neurodevelopmental conditions.

Beyond demonstrating effectiveness, the present findings suggest that embodied musical engagement operates as a developmental scaffold rather than a discrete therapeutic technique. For children with autism spectrum disorder and other developmental disabilities, bodily engagement with music appears to provide a structured yet flexible developmental environment in which emerging non-cognitive capacities can be activated, stabilized, and functionally expressed. This perspective extends existing music therapy literature by positioning embodied musical engagement not only as an intervention input, but as an organizing context that supports gradual developmental change under conditions of limited attentional, regulatory, and social resources.

First, the results indicated that embodied musical engagement substantially boosts non-cognitive skills (*β* = 0.755). This finding is consistent with embodied cognition theory, which suggests that cognitive, emotional, and social abilities are highly dependent on bodily activities, rhythmic synchronization, and sensory interactions (Srinivasan and Bhat, 2013). Rhythmic movements, body swaying, action imitation, and interactive music games within music activities provide structured, repetitive practice that can contribute to gradual improvements in behavioral regulation, the expression of emotions, and the duration of attentive engagement.

The results suggest that embodiment is a key mechanism through which music interventions produce cognitive and socio-emotional benefits, consistent with prior research showing that rhythm synchronization facilitates social behaviors and music activities activate social brain networks (Sharda et al., 2018).

Second, non-cognitive skills were found to have a significant positive effect on rehabilitation outcomes (β = 0.364). Non-cognitive skills, including emotional regulation, executive functioning, and cooperative behaviors, are closely related to rehabilitation goals such as functional learning participation, daily adaptation, social functioning, and classroom engagement. Children with more stable emotions and stronger attention regulation were more likely to remain engaged across rehabilitation activities, leading to progressive behavioral improvement. This finding corroborates the developmental and educational psychology literature, which highlights the central role of non-cognitive skills in children’s growth, and supports the special education emphasis on social–emotional competencies over purely academic skills.

Third, the structural model supports a dual-pathway model of music-based rehabilitation, in which embodied musical engagement influences rehabilitation outcomes through two complementary mechanisms. One pathway operates indirectly through the cultivation of non-cognitive skills, enhancing children’s capacity for attention regulation, emotional stability, and task persistence during rehabilitation activities. The second pathway operates more directly, through immediate sensorimotor activation, behavioral engagement, and motivational processes inherent in embodied musical experiences such as rhythmic synchronization and action-based participation. Together, these findings indicate that embodied musical engagement facilitates rehabilitation both by strengthening foundational developmental capacities and by supporting moment-to-moment functional participation.

Finally, all structural paths were significantly positive, demonstrating the explanatory power of the proposed theoretical framework. This evidence validates and broadens understanding of the links among music therapy, embodied interventions, and non-cognitive psychological development. Importantly, the present findings should be interpreted through a functional accessibility perspective rather than a performance optimization lens. For children with autism spectrum disorder and related developmental conditions, partial engagement, brief persistence, and intermittent regulation constitute meaningful developmental achievements rather than incomplete performance. Accordingly, the observed effects reflect improvements in children’s accessibility to rehabilitation demands, rather than attainment of optimal or age-normative performance levels. This perspective aligns with developmentally sensitive and inclusive approaches to rehabilitation research.

### 5.1 Managerial/practical implications

Based on the findings, the study provides the following practical recommendations for rehabilitation centers, special education schools, and policy-makers:

Integrate embodiment principles systematically into the design of the rehabilitation course (Geretsegger et al., 2022). Rehabilitation programs should go beyond passive listening to emphasize rhythmic synchronization and interactive movements. Designing tasks with predictable rhythms (e.g., clapping to a steady beat, imitating simple gestures) can lower the threshold for engagement, thereby promoting children’s attention and participation.
Develop non-cognitive skills–oriented rehabilitation assessment system. Current assessments often overlook the “soft skills” that drive success. Emotional regulation, attention control, task persistence, and cooperative behavior should be formally incorporated into rehabilitation records. This supports a more comprehensive and empirically grounded evaluation of therapeutic effectiveness, shifting focus from mere task completion to the development of internal regulatory capacities.
  Promote interdisciplinary collaborative rehabilitation models. Since non-cognitive skills are central to rehabilitation success, teams integrating music therapists, psychologists, and special education teachers can provide multidimensional interventions. This collaborative approach helps stabilize children’s behaviors and strengthen overall rehabilitation outcomes by addressing both physiological and psychological needs simultaneously (Kalas, 2012).

### 5.2 Limitations

There are several notable limitations of this study that should be acknowledged. First, this study adopted a cross-sectional observational design, which prevents rigorous causal inference regarding the temporal sequence of research variables. Although this design ensures high ecological validity by capturing natural and real-time relational snapshots during multicenter ongoing rehabilitation, it cannot rule out the possibility of reverse or bidirectional relationships between variables. For instance, children with superior non-cognitive skills may be inherently more willing to engage actively in musical embodied activities. Furthermore, cross-sectional data fail to fully capture the delayed and cumulative effects of embodied music engagement, as the cultivation of non-cognitive skills and rehabilitation progress develop dynamically over time. Therefore, future studies can adopt longitudinal follow-up designs to explore whether the strength of mediating pathways varies with intervention duration and verify the dose–response relationship of music-based rehabilitation interventions (Thompson et al., 2014; Yum et al., 2024). Second, this study primarily focused on the core direct and mediating effects of variables without systematic investigation of potential moderating factors. At the individual level, children of different age groups or diagnosed with distinct neurodevelopmental disorders (e.g., ASD vs. ADHD) may demonstrate heterogeneous responses to musical embodied engagement. At the contextual and environmental level, ecological factors including family support, socioeconomic status, and rehabilitation environment quality can significantly moderate the translation efficiency of musical participation into developmental and rehabilitation benefits. Future research could incorporate these individual characteristics and comprehensive ecological contextual variables to construct a more holistic theoretical framework, providing empirical evidence for personalized, context-sensitive, and precise rehabilitation intervention strategies. Third, limitations pertaining to measurement approaches require elaboration. While multiple statistical validation procedures, including the Fornell–Larcker criterion, model comparison, and the CLF method, confirmed the discriminant validity of all latent constructs in this study, all primary data were sourced from subjective reports of caregivers and therapists. In authentic clinical rehabilitation scenarios, children’s embodied engagement behaviors and non-cognitive performance are highly intertwined and difficult to differentiate merely through subjective observation and evaluation. To address this gap, future studies should employ a multi-method triangulation approach by integrating objective behavioral assessments (e.g., motion capture technology for rhythmic synchronization measurement) and physiological biomarkers (e.g., EEG and heart rate variability). Such multi-modal measurements can precisely quantify and disentangle the independent and unique predictive mechanisms of embodied engagement and non-cognitive skills on rehabilitation outcomes (Sharda et al., 2018).

## 6 Conclusion

This study moves beyond confirming the effectiveness of embodied music-based interventions by conceptualizing embodied musical engagement as a developmental scaffold that supports rehabilitation under conditions of limited regulatory and attentional capacity. Through a dual-pathway model, the findings demonstrate that music-based embodiment influences rehabilitation outcomes both indirectly, by strengthening non-cognitive developmental capacities, and directly, by facilitating moment-to-moment sensorimotor engagement and behavioral participation (LaGasse, 2017). By integrating embodied cognition theory with empirical evidence from structural modeling, this study provides a more precise and developmentally sensitive account of *how* and *why* music-based interventions support rehabilitation outcomes in children with special needs.

## Data Availability

The original contributions presented in the study are included in the article/Supplementary material, further inquiries can be directed to the corresponding author.
