# Supplementary material for: The role of embodied musical engagement in enhancing non-cognitive skills and rehabilitation outcomes in children with special needs
Source: Front Psychol. 2026 May 25;17:1798176. doi: 10.3389/fpsyg.2026.1798176 (PMC13243244; doi:10.3389/fpsyg.2026.1798176)
Supplement: Supplementary file 1 [file Data_Sheet_1.PDF]

The Role of Embodied Musical Engagement in Enhancing Non-Cognitive Skills and Rehabilitation Outcomes in Children with Special Needs

Table of Contents

Appendix A: Operationalization of Variables

Appendix B: Measurement Scale Items

- B1: Embodied Musical Engagement Scale
- B2: Non-cognitive Skills Scale
- B3: Rehabilitation Performance Scale

Appendix C: Integrated Survey Questionnaire (For Data Collection)

Appendix A: Operationalization of Variables

This study involves three core constructs: Embodied Musical Engagement (EME), Non-cognitive Skills (NCS), and Rehabilitation Performance (RP). The following table clarifies the definition, measurement dimensions, and the oretical sources of each construct.

Table A1. Operationalization of Constructs

Construct	Definition	Dimensions	Theoretical Sources
**Embodied Musical Engagement (EME)**	The degree to which children participate physically, rhythmically, and interactively in music-based activities, involving sensorimotor coupling and bodily expression.	1. **Rhythmic Entrainment** 2. **Physical Engagement** 3. **Imitative Action** 4. **Interactive Participation**	Leman (2007); Phillips-Silver &Trainor (2005); Srinivasan & Bhat (2013)
**Non-cognitive Skills (NCS)**	A set of emotional, attentional, motivational, and social abilities that support task persistence, self-regulation, and social interaction in rehabilitation contexts.	1. **Emotion Regulation** 2. **Attention Control** 3. **Task Persistence** 4.**Cooperative Behavior**	Heckman & Kautz (2012); Gutman & Schoon (2016); Blair & Raver (2014)
**Rehabilitation Performance (RP)**	The degree of improvement and engagement children demonstrate in rehabilitation training, including participation quality, behavioral stability, and functional gains.	1.**Engagement** 2.**Persistence** 3. **Behavioral Improvement** 4. **Social Skills**	Sharda et al (2018); Geretsegger et al. (2014) ; Yum et al. (2024)

Appendix B: Measurement Scale Items

This study uses a 5-point Likert Scale to measure all variables. All items are rated by the child's primary caregiver (parent/rehabilitation therapist) based on observations over the past month.

Rating Scale: - 1 = Never - 2 = Rarely - 3 = Sometimes - 4 = Often - 5 = Always

B1: Embodied Musical Engagement Scale (EME)

Dimension Description: This scale contains 4 dimensions with 3 items each, totaling 12

items.

Table B1. EME Scale Items

Dimension	Item Code	Item Content
**1. Rhythmic Entrainment**	EME_ENT1	The child can sometimes attempts to synchronize their movement to the rhythm of music.
	EME_ENT2	The child shows some rhythmic bodily responses during musical activities.
	EME_ENT3	The child notices and shows some response to tempo changes in music.
**2. Physical Engagement**	EME_PHY1	The child displays body movements such as clapping,tapping,or swaying during music.
	EME_PHY2	The child shows partical physical engagement rather than being entirely passive.
	EME_PHY3	The child attempts to use bodily movement in response to music.
**3. Imitative Action**	EME_IMI1	The child occasionally attempts to imitate teacher's simple movements during musical activities.
	EME_IMI2	The child observes others' gestures and sometimes attempts to replicate them.
	EME_IMI3	Imitative behaviors emerge more as the child participates in more sessions.
**4. Interactive Participation**	EME_INT1	With guidance, the child can participate in simple turn-taking interactions.
	EME_INT2	The child shows awareness of others' actions and occasionally responds.
	EME_INT3	With encouragement, the child can initiate simple interactions (e.g., offering an instrument).

SPSS Variable Naming Recommendation: Use item codes directly (e.g., EME*ENT1, EME*ENT2) as SPSS variable names for subsequent Confirmatory Factor Analysis (CFA)
.

B2: Non-cognitive Skills Scale (NCS)

Dimension Description: This scale contains 4 dimensions with 3 items each, totaling 12 items.

Table B2. NCS Scale Items

Dimension	Item Code	Item Content
**1. Emotion Regulation**	NCS_ER1	The child can gradually calm down with soothing after frustration during activities.
	NCS_ER2	The child is beginning to learn to express emotions appropriately rather than through tantrums.
	NCS_ER3	The child emotional reactions during structured tasks are relatively predictable.
**2. Attention Control**	NCS_AC1	The child can maintain brief focus during short tasks.
	NCS_AC2	The child can sometimes continue activities amid some distractions.
	NCS_AC3	After distraction, the child can reorient attention to the task with prompting.
**3. Task Persistence**	NCS_TP1	The child completes short activities with support.
	NCS_TP2	The child shows some willingness to try challenging tasks repeatedly.
	NCS_TP3	The child can remain engaged for a short while even when tasks become difficult.
**4. Cooperative Behavior**	NCS_CB1	Given clear instructions ,the child can follow basic activity group rules.
	NCS_CB2	The child shows willingness for simple cooperate with peers or teachers.
	NCS_CB3	The child can participate in simple activities that require joint engagement.

B3: Rehabilitation Performance Scale (RP)

Dimension Description: This scale contains 4 dimensions with 3 items each, totaling 12 items.

Table B3. RP Scale Items

Dimension	Item Code	Item Content
**1. Engagement**	RP_ENG1	The child follows rehabilitation tasks and occasionally shows interest.
	RP_ENG2	The child displays calms or positive emotional states during training sessions.
	RP_ENG3	The child shows preference or anticipation for specific rehabilitation activiies.
**2. Persistence**	RP_PER1	The child can remain present and engaged for most of a single session.
	RP_PER2	The child attempts adapted activities and shows effort.
	RP_PER3	The child shows gradual adaptation to training durations, with reduced withdrawal behaviors.
**3. Behavioral Improvement**	RP_BEH1	The child shows initial improvement in motor coordination within the training context.
	RP_BEH2	Compared to pre-training, the child's behavior in specific contexts is more stable.
	RP_BEH3	The frequency of challenging behaviors during structured tasks has decreased.
**4. Social Skills**	RP_SOC1	The child shows brief eye contact or orienting attention during interactions.
	RP_SOC2	The child can initiate basic communication through gestures or simple vocalizations.
	RP_SOC3	The child can respond to direct social cues from others(e.g., being called by name)

Appendix C: Integrated Survey Questionnaire (For Data Collection)

Title: Music Rehabilitation Effectiveness Survey for Children with Special Needs

Instructions

Dear Parent/Caregiver:

This questionnaire aims to understand the mechanisms through which music training affects rehabilitation outcomes for children with special needs. Your responses are completely anonymous and will be used solely for academic research. Please rate the child's performance over the past month. The questionnaire takes approximately 10-15 minutes to complete.

Rating Scale: - **1** = Never - **2** = Rarely - **3** = Sometimes - **4** = Often - **5** = Always

Part I: Basic Information

Please complete the following information:

1. **Your role:**
    - [ ] Parent
    - [ ] Grandparent
    - [ ] Rehabilitation Therapist
    - [ ] Primary Caregiver
    - [ ] Other: ___________
2. **Child's age:**
    - [ ] Under 6
    - [ ] 6-12 years
    - [ ] 13-18 years
    - [ ] Above 18
3. **Child's gender:**
    - [ ] Male
    - [ ] Female
4. **Primary disability type:**
    - [ ] Autism Spectrum Disorder
    - [ ] ADHD
    - [ ] Intellectual/Developmental Delay
    - [ ] Hearing Impairment
    - [ ] Physical Disability
    - [ ] Emotional/Psychological Disorder
    - [ ] Other: ___________
5. **Total duration of music rehabilitation training:**
    - _____ years _____ months
6. **Frequency of music training:**
    - [ ] Once per week
    - [ ] 2-3 times per week
    - [ ] 4+ times per week

Part II: Embodied Musical Engagement

Please rate the child's actual performance during musical activities:

No.	Item	1<br>Never	2<br>Rarely	3<br>Sometimes	4<br>Often	5<br>Always

**Rhythmic Entrainment**						
1	The child can sometimes attempts to synchronize their movement to the rhythm of music.	○	○	○	○	○
2	The child shows some rhythmic bodily response during musical activities.	○	○	○	○	○
3	The child notices and shows some response to tempo changes in music.	○	○	○	○	○
**Physical Engagement**						
4	The child displays body movements such as clapping, tapping, or swaying during music.	○	○	○	○	○
5	The child shows partial physical engagement rather than being entirely passive.	○	○	○	○	○
	The child					

6	attempts to use bodily movement in response to music.	○	○	○	○	○
**Imitative Action**						
7	The child occasionally attempts to imitate the teacher's simple movements during musical activities	○	○	○	○	○
8	The child observes others’ gestures and sometimes attempts to replicate them.	○	○	○	○	○
9	Imitative behaviors emerge more as the child participates in more sessions .	○	○	○	○	○
**Interactive Participation**						
10	With guidance, the child can participate in simple turn-taking interactions.	○	○	○	○	○
11	The child shows awareness of others actions and occasionally responds.	○	○	○	○	○

12	With encouragement, the child can initiate simple interactions(e.g. offering an instrument).	○	○	○	○	○

Part III: Non-cognitive Skills

Please rate the child's performance during daily rehabilitation training:

No.	Item	1 Never	2 Rarely	3 Sometimes	4 Often	5 Always
**Emotion Regulation**						
13	The child can gradually calm down with soothing after frustration during activities.	○	○	○	○	○
14	The child is beginning to learn to express emotions appropriately rather than solely through tantrums.	○	○	○	○	○
15	The child's emotional reactions during structured tasks are relatively predictable .	○	○	○	○	○

**Attention Control**						
16	The child can maintain brief focus during short tasks.	○	○	○	○	○
17	The child can sometimes continue activities amid some distractions.	○	○	○	○	○
18	After distraction, the child can reorient attention to thetask with prompting.	○	○	○	○	○
**Task Persistence**						
19	The child completes short activities with support.	○	○	○	○	○
20	The child shows some willingness to attempt challenging tasks repeatedly. .	○	○	○	○	○

21	The child can remain engaged for a short while even when tasks become difficult.	○	○	○	○	○
**Cooperative Behavior**						
22	Given clear instructions, the child can follow basic group activity rules .	○	○	○	○	○
23	The child shows willingness for simple cooperate with family peers or teachers.	○	○	○	○	○
24	The child can participate in simple activities that require joint engagement.	○	○	○	○	○

Part IV: Rehabilitation Performance

Please rate the child's overall performance in rehabilitation training:

No.	Item	1 Never	2 Rarely	3 Sometimes	4 Often	5 Always
**Engagement**						
25	The child follows rehabilitation tasks and occasionally shows interest.	○	○	○	○	○

26	The child displays calms or positive emotional states during training sessions..	○	○	○		○	○
27	The child shows preference or anticipation for specific rehabilitation activities.	○	○	○		○	○
**Persistence**							
28	The child can remain present and engaged for most of a single session.	○	○	○		○	○
29	The child attempts adapted activities and shows effort.	○	○	○		○	○
30	The child shows gradual adaptation to training duration, with reduced withdrawal behaviors.	○	○	○		○	○
**Behavioral Improvement**							
31	The child shows initial improvement in motor coordination within the training context.	○	○	○		○	○

32	Compared to pre-training, the child's behavior in specific contexts is more stable.	○	○	○	○	○
33	The frequency of challenging behaviors during structured tasks has decreased.	○	○	○	○	○
**Social Skills**						
34	The child shows brief eye contact or orienting attention during interactions.	○	○	○	○	○
35	The child can initiate basic communication through gestures or simple vocalizations.	○	○	○	○	○
36	The child can respond to direct social cues from others(e.g., being called by name)	○	○	○	○	○

End of Survey
Thank you for your participation! Your responses will be used solely for academic research purposes.
